# Adding predictive and diagnostic values of pulmonary ground-glass nodules on lung cancer *via* novel non-invasive tests

**DOI:** 10.3389/fmed.2022.936595

**Published:** 2022-08-18

**Authors:** Yizong Ding, Chunming He, Xiaojing Zhao, Song Xue, Jian Tang

**Affiliations:** ^1^Department of Thoracic Surgery, Renji Hospital, Shanghai Jiao Tong University School of Medicine, Shanghai, China; ^2^Department of Cardiovascular Surgery, Reiji Hospital, Shanghai Jiao Tong University School of Medicine, Shanghai, China

**Keywords:** ground-glass nodules, lung cancer, prediction, diagnosis, biomarker

## Abstract

Pulmonary ground-glass nodules (GGNs) are highly associated with lung cancer. Extensive studies using thin-section high-resolution CT images have been conducted to analyze characteristics of different types of GGNs in order to evaluate and determine the predictive and diagnostic values of GGNs on lung cancer. Accurate prediction of their malignancy and invasiveness is critical for developing individualized therapies and follow-up strategies for a better clinical outcome. Through reviewing the recent 5-year research on the association between pulmonary GGNs and lung cancer, we focused on the radiologic and pathological characteristics of different types of GGNs, pointed out the risk factors associated with malignancy, discussed recent genetic analysis and biomarker studies (including autoantibodies, cell-free miRNAs, cell-free DNA, and DNA methylation) for developing novel diagnostic tools. Based on current progress in this research area, we summarized a process from screening, diagnosis to follow-up of GGNs.

## Introduction

Pulmonary ground-glass nodules (GGNs) with a ground-glass opacity (GGO) are radiological findings on computed tomography (CT) images (1). GGNs are consisted of hazy lesions with increased lung attenuation that usually do not obscure underlying bronchial structures or pulmonary vessels (2). With the widespread use of high-resolution CT imaging, GGNs are increasingly detected in both benign and malignant conditions such as focal interstitial fibrosis, inflammation, hemorrhage, and cancer (3). Accumulated evidence has suggested that GGNs are well associated with lung cancer. Slow-growing or stable GGNs often indicate the presence of early-stage lung cancers or preinvasive lesions. The extent of invasiveness of GGNs is an important prognostic indicator in lung adenocarcinoma, as suggested by multivariate analysis (4, 5). GGN has been described as a special subtype of lung cancer that are different from conventional lung adenocarcinoma, therefore, the management of GGNs should be different from that of lung cancer with solid nodules. In 2017, the Fleischer society released the second major guidelines on the management of GGNs, which was based on the type and size of GGNs as well as patient conditions and preference. Accurate prediction, early diagnosis along with optimized therapeutic approaches and follow-up plans are all involved in a comprehensive strategy for clinical management of GGNs. Here, we reviewed radiologic and pathological characteristics of different types of GGNs as well as recently developed diagnostic tools, then subsequently summarized a process involving identification of patients or high-risk individuals, prediction and diagnosis of GGNs, as well as long-term follow-up strategies.

## Classification of ground-glass nodules

GGNs are classified into pure GGN (pGGN), heterogenous GGN (hGGN) and part-solid GGN. Heterogenous and part-solid GGNs are often called mixed GGN (mGGN). Notably, GGN is often a focal finding that represents lung infections, lung edema with fluid in the interstitium, patchy increased parenchymal perfusion, or interstitial diseases where GGN precede irreversible change. Around 40–50% of pGGN and hGGN regressed or disappeared within 3 months, indicating the inflammatory nature of these GGNs (6). The management plans of pre-invasive and invasive lesions are different, recent studies on GGNs have made a significant contribution to understand the pathophysiology of GGNs and improved the diagnostic accuracy.

### Pure ground-glass nodule

Although pGGN contains no visible solid component in both the lung and mediastinal windows, several studies showed that more than 50% of pGGN showed growth with favorable prognosis and only 1.2% of pGGN can develop into mGGN (7–10). The average time for pGGN to develop into mGGN was 3.8 ± 2.0 years (11). Many international guidelines have adopted the treatment for pGGN.

### Heterogenous ground-glass nodules and part-solid ground-glass nodules

hGGN denotes a nodule with a visible solid component in the lung window but not in the mediastinal window, whereas part-solid GGN refers to a nodule with a visible solid component and present in both the lung and mediastinal windows (12). mGGN appears to be more invasive than pGGN. The average time for hGGN to develop into a part-solid nodule was 2.1 ± 2.3 years (11), and the sizes of these nodules tend to be the indicator of invasiveness.

## Benign vs. malignant ground-glass nodules

### Computed tomography features

Although pulmonary GGNs have a high probability of being malignant, some benign nodules have been misdiagnosed as lung cancer and treated by unnecessary surgical resection. Thus, it is important to have these GGNs well-characterized prior to the decision-making of therapeutic strategies. Previous studies failed to characterize the common features of benign GGNs (13, 14). Furthermore, although the cut-off value of 6 mm for GGN was used to predict the pathological invasiveness (15, 16), it remains unclear the optimal cut-off value of solid portion on CT that correlates to pathological invasive components. Several studies have characterized GGNs based on their CT manifestations to find a clue for the differentiation between benign and malignant GGNs (Table 1).

**TABLE 1:** The characteristics of benign and malignant GGNs.

Pathological type	Size	GGN type	Morphological findings	Clinical staging	References
Benign	< 30 mm	pGGN	Ill-defined single isolated nodules; high-attenuation zone with blurred edge; may connect to adjacent blood vessel	N/A	Li et al. (17)
AAH	< 5 mm	pGGN	Mildly to moderately atypical type II or Clara cells along alveolar walls, alveolar septa, or respiratory bronchiole	N/A	Mori et al. (21)
AIS	< 30 mm with solid part	pGGN or mGGN	Lepidic growth with neoplastic cells along the alveolar structures; increased intensity; no stromal, vascular, or pleural invasion	pTis	Travis et al. (22)
MIA	< 30 mm with solid part < 6 mm	mGGN	Pure or predominant lepidic growth with neoplastic cells along the alveolar structure; no lymphatic, vascular, or pleural invasion; no tumor necrosis; no spread through alveolar air spaces	pT1Mi	Gardiner et al. (23)
IVA	> 30 mm with solid part > 6 mm	mGGN	Regular well-defined nodule; irregular and multiple solid components with coarse margin; air bronchogram with disruption, irregular dilation, and pleural indentation	pT1a-T1c, pT2-4	Yanagawa et al. (25)

AAH, atypical adenomatous hyperplasia; AIS, adenocarcinoma in situ; IVA, invasive adenocarcinoma; MIA, minimally invasive adenocarcinoma; mGGN, mixed ground-glass nodule; pGGN, pure ground-glass nodule.

The characterization of CT features of benign nodules was reported in a recent retrospective study on 79 patients with GGNs. Some common CT manifestations were identified, including a regular or irregular nodule with an isolated, blurred internal high-attenuation zone connecting to the blood vessels, or a nodule abutting but not surrounding blood vessels, suggesting that follow-up should be firstly considered for GGNs with these characteristics for a higher likelihood of benignity (17).

Larger pulmonary nodules are more likely to be malignant than nodules with smaller sizes, however, the solid components are often irregular with coarse margins and present as multiple spots. Importantly, the blood vessels passing through malignant GGNs were usually distorted or dilated, whereas the blood vessels were not affected inside benign GGNs (18, 19). According to the 4th edition of WHO lung cancer classification in 2015, lung adenocarcinoma can be classified into preinvasive and invasive lesions. The preinvasive lesions, including atypical adenomatous hyperplasia (AAH) and adenocarcinoma *in situ* (AIS), may progress to minimally invasive adenocarcinoma (MIA) and finally to invasive adenocarcinoma (IVA) (20). Most AAH, AIS, and MIA are pGGNs, which grow along respiratory bronchiolar wall and alveolar wall (21–23). If there are obvious local fibroblast proliferation, local accumulation of tumor cells or alveolar wall collapse, then AAH, AIS, and MIA could represent as mGGNs. A study using multi-detector CT indicated that the size of the whole nodule (≥ 9.8 mm) and its solid component (≥ 0.9 mm), as well as the mixed GGN nature of the nidus, burr and lobes can all distinguish IVA and pre-invasive lesions (24). To further differentiate IVA and AIS, Yanagawa et al. showed some morphological differences between the two (25). Both AIS and IVA displayed a feature of inflammatory nodules with spiculated margin, which might be due to the accumulation of mucin in the surrounding normal alveolar space, however, IVA tends to show external or internal convex with the spiculated margin, and often with a solid portion > 7.3 mm on thin-section CT (TSCT) (25). Moreover, the air bronchogram with disruption and irregular dilatation were more likely appeared in IVA than AIS (25). In addition, GGNs in lung cancer become lobulated due to desmoplastic reaction (26).

In addition to nodular size, the association between number of nodules and malignancy has also been studied previously. In the NELSON trial on 3,392 participants for investigating the relationship between number of nodules and the probability of lung cancer, it showed that 51.5% participant had one nodule, 23.6% had two nodules, 10.4% had three nodules, 5.6% had four nodules, and 8.9% had more than four nodules (27). Thus, almost half of the participants showed multifocal pulmonary GGNs. However, the probability of lung cancer among these participants was 3.6% for one nodule, 4.1% for two nodules, 4.8% for three nodules, 6.3% for four nodules, and 3.3% for > 4 nodules (27). Thus, the number of nodules was not a good indicator for lung cancer.

### Risk factors contributing to malignant ground-glass nodule

The etiology of GGN is largely unknown, however, smoking and aging are considered as major risk factors contributing to the development of GGN (28, 29). Tobacco consumption is implicated in 85% of all cancer-related deaths (30). The duration of active smoking is much more relevant than the number of cigarettes smoked per day in estimating the risk of lung cancer (31). Smoking history is one of the predictors of GGN growth and its invasive extent (32). Also, a higher frequency of the appearance of GGN was observed in smokers than in non-smoker over a mean period of 5.5 years (33). More importantly, in the last decade, increased evidence has been provided to show that smoking cessation plus regular annual chest CT screening may reduce the risk of lung cancer (34–36). While it remains unclear whether smoking cessation after diagnosis of GGNs alters the clinical behavior of GGNs, smoking cessation could have substantial benefits for patients with GGNs.

Aging is another principal risk factor for GGNs. Lung cancer was developed above the age of 70 in more than half of the cases (37). Aging seemed to contribute to a higher probability of malignancy in individuals with GGNs, however, age cannot be used as a single predictor for predicting the pathological feature or invasiveness of GGNs (38). For instance, GGNs were accidently detected in the lung of 12 suspected COVID-19 teenager patients in China when performing CT scan, which turned out to be MIA in 10 teenagers and IVA in two (39). However, the reason underlying the development of GGNs in teenagers remain unclear. Smoking and family history are considered as minor risk factors in teenage patients (39), whereas other factors such as exposure to second-hand smoke or air pollution may explain the incident of GGN in these patients. However, further research on the amount and duration of exposure to these potential carcinogenic factors may shed the light on the understanding of pathogenesis of GGNs, as well as the strategy making for primary prevention and early diagnose of GGN-associated lung cancers.

## Novel approaches for diagnosis of ground-glass nodules

Imaging scans and specific biomarkers alone or in combination have been tested for their potential and accuracy of detection, prediction and diagnosis of GGNs. Further studies are required to understand whether these tests are sufficient to provide diagnostic accuracy on differentiation of various types of GGNs and their invasiveness. The diagnostic value of these non-invasive tests will need to be confirmed by large-scaled studies to get approval for clinical use.

### Imaging scans and biopsy

To date, the detection of GGN or lung cancer still rely heavily on imaging techniques such as CT or MRI (40). To enhance the diagnostic accuracy of invasive extent of GGN, a multifactorial radiomics model that selects quantitative imaging features extracted from CT images has been developed to create unique fingerprints of these images, which can be used in combination with frozen section results and clinical data to evaluate the pathological classification of peripheral lung adenocarcinoma manifesting as IVA from MIA (41, 42). Importantly, if this combined method can provide a quick result on pathology, a curative resection can be performed in the same operation, which may make substantial improvements on the management of GGNs. This CT-based radiomics model demonstrated that the diameter and type of nodules were significantly different between IVA and MIA, for smaller-sized pGGN increased the probability of MIA diagnosis (41). A more recent research using a combined nomogram with radiographic and radiomics features showed high diagnostic accuracy and efficacy of the type and invasiveness of GGN prior to surgery (43). However, this method requires to be further verified by larger-scaled randomized controlled trials (RCTs), with the help of better semiautomatic techniques separating GGNs from pleura or blood vessels. Nevertheless, imaging scans have shown some limitations due to unsatisfactory sensitivity and specificity in detecting malignancy of nodules and determining resection margins, therefore, alternative methods for single use or in combination with imaging techniques are urgently needed.

Positron emission tomography-computed tomography (PET-CT) is an imaging technique that has been utilized to metabolically categorize solitary pulmonary nodules (SPNs) (44). The overall sensitivity has been reported to reach 95%, and the specificity reported to reach 82% in non-oncologic cohorts (44). The technique has been utilized in discrimination of lung cancers and prediction of possible responsiveness to treatments. For instance, peak standardized uptake value (SUVpeak) has been reported to be utilized to predict early response for adaptive radiation in locally advanced non-small cell lung cancer (45). Recent advances in techniques have highlighted the utilization of PET-CT scanning to determine invasiveness of GGNs, and to predict probable response to certain interventions. For instance, one study has revealed that the proportion of IVA increased with elevation of maximum SUV (SUVmax) (46), and that SUV index independently predicted invasiveness of adenocarcinoma GGNs (47). In addition, a SUVmax of 2.0 has been proposed to differentiate IVA from AIS-MIA, and the growth pattern of the adenocarcinoma could also be differentiated when the SUVmax was set to 1.4 (48). Furthermore, another study evaluating adenocarcinoma with a GGO component less than 30 mm revealed that the mean SUVmax varied among types of adenocarcinoma (49). Together, these results provided critical information on the association between PET-CT and GGNs, suggesting future directions for utilization of PET-CT in differential diagnosis of GGNs.

For preoperative diagnosis, imaging techniques are often used in combination with transthoracic fine needle biopsy, which is less invasive than thoracic surgeries, and is an effective diagnostic approach for GGNs in experienced hands. However, inadequate sampling, false-negative results and risk of pneumothorax have limited its use in the diagnosis of very small or deep nodules and GGNs (50). Under such circumstances, the liquid biopsy is developed as a novel non-invasive approach for clinical diagnosis of pulmonary nodules (51).

### Biomarkers for preoperative diagnosis

The development of trustworthy biomarkers for the diagnosis of GGNs has never been ceased. Some of these biomarkers, including autoantibodies, cell-free miRNAs, cell-free DNA (cfDNA), and DNA methylation, were designed to be tested prior to operation for early prediction and detection. For the purpose of lung cancer diagnosis, the specimen used for liquid biopsy includes peripheral circulating blood, sputum, and bronchoalveolar lavage fluid which contains tumor cells and tumor-derived products released.

An autoantibody (AAb) array panel detecting seven AAbs (CAGE7, CAGE, MAGEA1, SOX3, GBu4-5, PGP9.5, and p53) in serum samples was developed for this purpose (52). Positive results from this AAb test panel were associated with a high risk of lung cancer with a high specificity (85.3%), a moderate sensitivity (45.5%) and a positive predictive value (72.7%) during the follow-up period (52). This panel has been suggested to be used in combination with CT for an early prediction of lung cancer, which may provide more accurate diagnosis of GGNs.

Furthermore, a recent study evaluated the diagnostic value and molecular characteristics of a non-invasive diagnostic tool testing plasma extracellular vesicles (EVs)-derived miRNAs for patients with GGNs (53). The distinct miRNA profiling (miR-500a-3p, miR-501-3p, and miR-502-3p) has been shown to accurately distinguish malignant and benign nodules with a specificity of 100% and a sensitivity of 98.9%, indicating that it may be helpful for clinicians (53). In another study, the plasma expression levels of miRNA-145a, miRNA-200b, and miRNA-7 in patients with early-stage non-small cell lung cancer (NSCLC) were significantly higher than those in patients with benign nodules (54). A prediction model combining these three miRNAs and CT features (pleural indentation and speculation) showed a sensitivity of 92.9% and a specificity of 83.3% in identification of early-stage lung cancer (54). Therefore, these specific miRNA panels may help clinicians to identify the nature of GGNs with a high diagnostic efficiency.

Small fragments of cfDNA are often released from apoptotic cells into blood, while in cancer patients, larger fragments are produced and released, thus, the ratio of shorter to longer cfDNA fragments, which is called cfDNA integrity, is changed (55). It was reported that patients with NSCLC had a significantly higher mean plasma level of cfDNA compared to those with benign nodules and healthy controls (56). When comparing to plasma level, the cfDNA integrity demonstrated a higher sensitivity (91% vs. 86.4%) and specificity (68.2% vs. 61.4%) in the discrimination of NSCLC and benign nodules (56). More recent research has developed a genome-wide approach to analyze cfDNA fragmentation profiles, providing a view of cfDNA “fragmentomes” which evaluates the size distribution of cfDNA fragments across genome and identify tumor-derives changes in cfDNA (57). This novel approach has shown a high performance in detecting early stage of lung cancer in high-risk smoking population (57), however, a large prospective validation will be needed before clinical use. Due to its potential in the early diagnosis of lung cancer, the plasma level, integrity and “fragmentomes” of cfDNA will need to be analyzed in patients with different types of GGNs in future studies.

Tumorigenesis can induce abnormal regulatory mechanisms such as DNA methylation (58). By using non-invasive sequencing targeted DNA methylation (called PulmoSeek) in plasma samples of patients with pulmonary nodules, a model was established and validated to predict the nature of GGNs ranging between 6 and 20 mm in size (59). It seemed to be a better diagnostic tool with a higher sensitivity and specificity to differentiate malignant GGNs from benign GGNs, as compared to PET-CT and mayo clinical model which combined clinical diagnosis and radiographic results (59). Another DNA methylation model with low-dose computed tomography (LDCT) screening is currently being developed by the same research group. Though effective, these diagnostic tools may require specialized DNA methylation test and analysis.

### Biomarkers for intraoperative diagnosis

Currently, intraoperative frozen sections are generally used for intraoperative diagnosis. Some biomarkers are being developed to test resected tissues obtained intraoperatively. For instance, an antibody array detecting the level of insulin-like growth factor-binding protein 2 (IGFBP2) and P-cadherin *via* semi-dry dot-blot method was developed for this purpose, based on the fact that high levels of these two proteins are indicators of micropapillary or solid components in early-stage lung cancer (60). The detection of micropapillary or solid components is particularly important for the determination of the extent of resection, however, is difficult to be fulfilled by traditional approaches including intraoperative frozen sections (60).

**TABLE 2:** Follow-up recommendations of GGNs from different guidelines.

Guideline	pGGN			Part-solid GGN*			Multiple sub-solid^#^
	Size^∧^	Malignant potential	Recommendation	Size^∧^	Malignant potential	Recommendation	Recommendation
2017 Fleischner guidelines	< 6 mm	Low	No routine follow-up unless suspicious or developed	< 6 mm	Low	No routine follow-up	CT at 3–6 months, then at 2–4 years, subsequent management based on the most suspicious nodule(s)
	≥ 6 mm	Low	CT at 6–12 months, then every 2 years until 5 years	≥ 6 mm, solid part < 6 mm	Low	CT at 3–6 months, then annual CT for 5 years	
				≥ 6 mm, solid part ≥ 6 mm	High	PET/CT, biopsy or resection is recommended	
2015 British thoracic society guidelines	Enlarge ≥ 2 mm		Consider (sublobar) resection/non-surgical treatment (SABR or RFA) or observation (repeat CT in 6 months)	enlarge solid component		Favor resection/non-surgical treatment over observation	For sub-solid nodules: reassess with CT at 3 months
	< 5 mm	Premalignant (AAH)		Solid area < 5 mm	Invasive (MIA)		< 5 mm: no routine follow-up.
	> 5 mm up to 30 mm	Premalignant (AIS)		Larger part-solid PSN	Invasive (adenocarcinoma)		≥ 5 mm, risk of malignancy < 10% (by Brock risk prediction tool), repeat CT at 1,2,4 years
							≥ 5 mm, risk of malignancy > 10%, discuss the options
2013 ACCP guidelines	≤ 5 mm	Mostly AAH or AIS, IVA is rare	No further evaluation	≤ 8 mm	Mostly AIS and IVA	CT at 3,12,24 months, then annual CT for an additional 1–3 years	Evaluate each nodule individually
	> 5 mm		Annual CT for at least 3 years	> 8 mm		Repeat CT at 3 months, then further evaluation with PET, biopsy, and/or surgical resection for nodules that persist	
	> 10 mm	Incidence of IVA is 10–50%	Follow-up at 3 months + biopsy and/or surgical resection for nodules that persist	> 15 mm		Further evaluation with PET, biopsy, and/or surgical resection	
2012 AATS guidelines	< 5 mm		Annual LDCT screening until age 79	< 5 mm		Annual LDCT screening until age 79	
	5–10 mm		LDCT in 6 months, if stable, annual LDCT screening until age 79, if suspicious change in size or appearance, surgical excision	5–10 mm		LDCT in 6 months, if stable, annual LDCT screening until age 79, if suspicious change in size or appearance, surgical excision	
	> 10 mm		LDCT in 3–6 months, if stable, LCDT in 6–12 months or biopsy or surgery; if suspicious change in size or appearance, surgical excision	> 10 mm		LDCT in 3–6 months, if stable, LCDT in 6–12 months or biopsy or surgery; if suspicious change in size or appearance, surgical excision	

^∧^Size refers to maximal diameter of nodule; *part-solid GGN refers to GGN with a solid component but > 50% ground glass; ^#^subsolid GGN includes pGGN and part-solid GGN. AAH, atypical adenomatous hyperplasia; AATS, The American Association for Thoracic Surgery; ACCP: American College of Chest Physicians; AIS, adenocarcinoma in situ; LDCT, low-dose computed tomography; MIA, minimally invasive adenocarcinoma; RFA, radiofrequency ablation; SABR, stereotactic ablative body radiotherapy.

### Genetic markers

Increasing efforts have been taken to understand the role of genetic alterations in the potential growth and pathological development of GGNs. Genetic analysis showed that mutation of epidermal growth factor receptor (*EGFR*) was the core signaling pathway in the development of GGN toward lung adenocarcinoma. For instance, Lee et al. identified a high frequency (89%) of *EGFR* mutations from 9 patients with GGNs (61). In a larger cohort, *EGFR* mutations were identified in 64% of 104 resected GGNs from 96 patients (62). Additionally, *EGFR*-mutation-positive GGNs were highly correlated with the growth of MIA/IVA when compared with *EGFR*-mutation-negative GGNs (62). Therefore, *EGFR* gene mutations have been identified as a dominant driver in the tumorigenesis of GGN lung adenocarcinoma.

The second critical gene that involves in the growth of GGNs is the Kristen rat sarcoma viral oncogene homolog (*KRAS*) gene. The *KRAS* mutation is the most common gain-of-function mutation, which was found in about 30% of lung adenocarcinomas in western countries (63) and approximately 8–13% in Asian cases (64, 65). For specific pathological types, *KRAS* mutation has been measured in 33%, 12%, 8%, and 0% of AAH, AIS, MIA, and well-differentiated adenocarcinoma samples, respectively (66). This gene encodes a membrane bound small GTPase and acts as an inducer for cell growth and division, however, mutation in *KRAS* impaired the ability to hydrolyze GTP and failed to lock the oncoprotein and activate *KRAS* downstream signaling cascade, leading to uncontrolled cell proliferation and survival. Animal and cell models have been used to further understand how *KRAS* mutations work in the carcinogenesis. Genetically modified mouse model demonstrated that not all *KRAS*-mutant lung cells equally developed to adenomas and malignant adenocarcinomas and only those derived from surfactant protein C + alveolar type II cells may progress to IVA (67, 68). The transcriptomic analysis of AAH in mouse demonstrated that a subset of cells displaying the signature of lung adenocarcinoma where others showed similar transcriptional profiling as normal alveolar cells (69, 70). Notably, *KRAS*-mutant lung adenocarcinomas showed specific pathological features and are associated with mucinous adenocarcinoma with goblet cell morphology (71). Overall, *KRAS* mutation could be used as an indicator for lung adenocarcinoma, especially in western populations.

Apart from *EGFR* and *KRAS*, the proto-oncogene B-Raf (*BRAF*) gene that encodes a serine/threonine kinase was identified in 3% (18/687) of western patients with lung adenocarcinomas (72) but only in 0.5% (25/5,125) of Asian patients (64). When analyzing resected samples, somatic *BRAF* variants have been reported in 23% of AAH samples, and highly correlated with *EGFR* mutations (73). However, the relevance of *BRAF* and *KRAS* mutations was decreased with the advance of lesion invasiveness, indicating that a subset of *BRAF*- or *KRAS*-mutated GGNs may undergo spontaneous regression.

Other mutations, including anaplastic lymphoma kinase (*ALK*) and human epidermal growth factor receptor 2 (*HER2*), have also been reported in patients with GGNs, with an incidence of 3 and 4%, respectively (62). Importantly, GGNs that are negative for *EGFR*, *BRAF*, *ALK*, and *HER2* was highly associated with AAH/AIS without growth of the nodules. When targeting exon sequencing and RNA sequencing from 9 individuals with either pGGN or part-solid GGN, in addition to *EGFR* and *BRAF*, other genes such as isocitrate dehydrogenase (NADP(+)) 2 (*IDH2*), tumor protein 53 (*TP53*), phosphatase and tensin homolog (*PTEN*) and EPH receptor B4 (*EPHB4*) were also identified as putative driver mutations of GGN adenocarcinomas (61). In the same study, the author also identified additional five novel fusion gene loci (*MED13L/TDRD3*, *SAMD12/TFA2*, *CEP250/TOP2A*, *TADA2A/MMP9*, and *TMEM243/DMTF1*), which were detected in relatively larger GGNs and could be associated with GGN progression, but not the initiation (61). With regards to *EPHB4*, a previous study targeting exon sequencing and RNA sequencing on GGN lung adenocarcinomas suggested that *EPHB4* gene mutations observed in patient with GGN was associated with cell proliferation and cellular motility in lung cancer (61). Moreover, although GGN lung adenocarcinoma was thought to be related to viral infection through a similar histology with ovine pulmonary adenocarcinoma caused by the Jaagsiekte sheep retrovirus (JSRV) in sheep (74), virus-associated transcripts have not been detected in GGNs, suggesting virus infection was not involved in the tumorigenesis of GGN lung adenocarcinoma (61).

Taken together, further studies utilizing genomic analysis on gene mutations in combination with functional analysis are required to elucidate the underlying signaling mechanisms governing the GGN progression to IVAs, also, further genetic analysis on different types of GGNs are required to confirm and identify potential genetic markers associated with GGN growth and progression, yielding the development of better management strategies for patients with GGN.

## Treatment, follow-up and clinical outcomes

A number of guidelines from the Fleischner Society, British Thoracic Society, American College of Chest Physicians (ACCP), and the American Association for Thoracic Surgery, have been published in recent years with follow-up recommendations included (Table 2) (75–79). A multidisciplinary specialist team, including pulmonologists, oncologists, radiologists, and thoracic surgeons are required for patients with lung nodules to predict the probability of malignancy and establish the management plan.

### Treatment of ground-glass nodules

Lobectomy and sublobar resections including wedge resection and anatomical segmentectomy are the standard surgical treatments for early-stage lung cancer, which have also been performed on GGN patients. For instance, when a curative surgical resection was provided as an initial treatment to a total of 171 hospital employees presented as GGN, no GGN recurrence or death was observed after a median follow-up period of 38 months (80). Lobectomy might be superior to sublobar resection for IVA, whereas sublobar resection is recommended for AAH, AIS, and MIA (81). Wedge resection performed on pGGNs has resulted in a 100% survival rate (82). In addition to these conventional open surgical approaches, video-assisted thoracoscopic surgery (VATS) is preferential to resect small pulmonary lesions and solitary GGNs with less invasiveness (79). Localizing methods such as radio-guided techniques have been proposed when pinpointing and removing small, subsolid or deep nodes by VATS, by which a 98% success rate and a 3.3% postprocedure pneumothorax rate has been reported by an Italian team having a 20-year experience with such techniques (44). For those who are not fit for surgery, non-surgical treatments may be considered, including stereotactic body radiation therapy (SBRT) and radiofrequency ablation (RFA).

In recent years, an increasing number of teenage lung cancers have been asymptomatically and incidentally diagnosed by CT scans. Characterized by small GGNs in radiology and pre-invasive or IVA in pathology, a good prognosis after wedge resection by VATS without recurrence in follow-up has been shown in 10 out of the 12 studied teenagers (39). The post-resection CT follow-up is suggested in a frequency of every 6 months for these teenage patients if the lesion progresses into IVA (83), and multidisciplinary discussion would be required (84). Due to lack of relevant research, more efforts need to be made to formulate a strategy for early diagnosis and early treatment in these teenage patients.

The scope of surgery for patients with multiple GGNs has always been controversial, and sublobar resection is generally considered as more appropriate than lobectomy for smaller GGNs and pGGNs. For ipsilateral synchronous multiple GGNs, the prognosis seems to be satisfactory after a single-stage surgical treatment (85). If multiple GGNs are in the contralateral chest, synchronous or two-stage VATS surgery can be implemented with satisfying outcomes (86). The condition of extremely multiple GGNs is defined as number of GGNs ≥ 3 that were surgically removed and pathologically diagnosed, with diameters of nodules between 3 and 30 mm. The prognosis of patients with extremely multiple GGNs was not affected by sublobar resection for pGGNs and non-main lesions (87), and the majority of patients did not experience an enlargement of unresected nodules during postoperative follow-up (87, 88), indicating that sublobar resection may be a priority for these patients. However, regarding the long-term outcomes, it was observed in a previous study that marginal or primary recurrence occurred in 4 out of 26 GGN patients 5 years after local resection (89). Therefore, in future studies, long-term follow-up should be carefully conducted for these patients after sublobar resection, and more clinical data regarding site, type, number and solid component of nodules needs to be accumulated, to recommend strategies of surgery, repeat of surgery and follow-up for multiple GGNs.

### Follow-up of ground-glass nodules

Most guidelines highly recommend an imaging reassessment within 3 months’ time after first detection of GGN with a size above 10 mm, while a reassessment within 6–12 months’ time is recommended for GGN above 5 mm. This is because mean resolve time of transient GGNs appears to be 4.8 months and most GGNs disappeared within 3 months (17). The Lung-RADS recommended that the strategy for follow-up of pGGN depends on the size of the nodules. For GGN < 30 mm or ≥ 30 mm but remains unchanged or grows slowly (Lung-RADS category 2), continue annual screening should be performed with LDCT in 12 months; For GGN ≥ 30 mm on baseline CT or new (Lung-RADS category 3), a 6-month LDCT should be performed.^1^ Part-solid and multiple sub-solid GGNs tend to be more frequently and carefully monitored, but the strategies for these follow-ups also depend on the size of these nodules, as suggested by Lung-RADS. Persistent GGN on CT can be suspected with a high risk of malignancy. Patient who underwent surgical or non-surgical treatment are recommended to be followed up every 3 months after the operation for 2 years with CT scanning every 3–6 months.

### Clinical outcomes

pGGN seems to have a good prognosis more frequently than other types of GGNs. A study by Cho et al. showed that patients with pGGN had a 100% survival rate and showed no lymph node metastasis over a mean observation period of 47.9 months (90). Although pGGN is not considered as a significant predictor of malignancy, solid component has been observed during follow-up period in studies of patients with pGGN (91–93). The average size of pGGN was less than 10 mm (9), therefore, nodular size was not regarded as an independent predictor for invasiveness of GGN (9, 94).

For part-solid GGNs, a cut-off value of 0.5 has been proposed for consolidation-to-tumor (CTR) ratio (95). CTR was useful to classify part-solid GGN and assist in the determination of surgical strategies. For instance, GGNs with a CTR below 0.5 has been shown to have a higher prevalence of lymph node involvement and a reduced recurrence-free survival. For sub-solid GGNs (including pGGN and part-solid GGN) within a range of 2.0 cm in maximal diameter, lung volume-preservation surgery, segmentectomy and wedge resection are preferred. Pathological analysis of the most progressive nodules showed stage 0/IA1 in 101 cases (59.1%), IA2/IA3 in 49 cases (28.7%), and IB or above in 21 cases (12.3%). Results of surgery was evaluated and showed 95.2% for 5-year relapse-free survival and 93% for overall survival (96). Thus, the best strategy to treat multiple GGN was to focus on invasive nodules that were solid or sub-solid.

## Conclusion and future research direction

Being one of the most commonly diagnosed cancers and with relatively low survival following diagnosis, lung cancer has received increasing attention. The trends for incidence of lung cancer vary between males and females, with the incidence decreased in males, and increased in females in multiple European countries (97). Though the mortality rate has been shown to decrease constantly from 2000 to 2017 in Northern America and Europe based on WHO Mortality Database, a significant increase of mortality has been reported by multiple countries (98). Therefore, diagnosis and interventions at the earliest stage are still warranted to facilitate optimal outcomes., Lung cancer, especially at its early stage, can present as GGNs on CT images. Not all GGNs grow or develop into invasive malignancies, however, slow-growing nodules or nodules persist often indicate the presence of early-stage lung cancers. Current critical challenges for treating physicians are to determine whether GGNs will progress and how to make optimal therapeutic strategies for patients to yield a better outcome.

Recent clinical and genetic data elucidate the pathophysiological aspect of different pathological types of GGNs, including benign, AAH, AIS, MIA, and IVA. In addition to traditional pathological evidence gained from biopsy specimens and intraoperative frozen sections, genetic analysis and novel serum biomarkers were under development to provide further evidence of malignancies with less invasiveness of procedures. Autoantibody array panel, EV-derived miRNAs, cfDNA analysis, and DNA methylation have attracted attention, as they provide additional information to facilitate differentiation of GGN types. More importantly, the use of these biomarkers in combination with imaging techniques may form optimal prediction models for the diseases. Although still at early stage for clinical use, these candidates have shown potentials as disease biomarkers, which requires further investigations.

The Fleischer society and other major societies of this field have recommended management of GGNs in their guidelines in the past decade, in which therapeutic and follow-up strategies are made based on size, solid component and type of GGNs, with patients’ conditions and preference being taken into consideration as well. However, inconsistent recommendations have been noticed in these guidelines, e.g., the cut-off value of nodular size, the follow-up intervals and methods, which need to be validated in large-scaled RCTs for certain populations.

**FIGURE 1:**
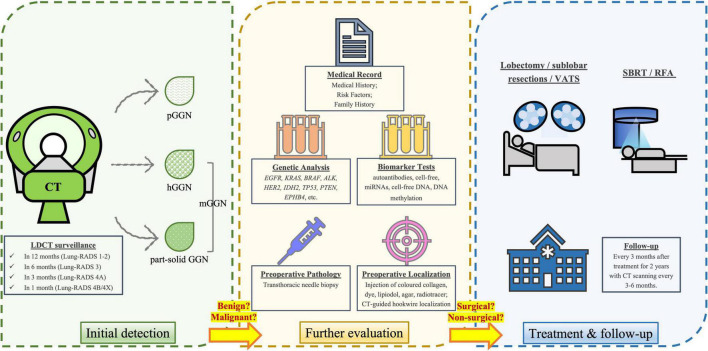
Diagnostic, treatment and follow-up procedures for individuals with GGNs. After initial detection *via* LDCT scan, a repeat LDCT in 12 months is recommended for negative findings or benign appearance/behavior (Lung-RADS category 1-2), while a 6-month repeat of LDCT is recommended for those with probably benign nodules (Lung-RADS category 3). The suspicious nodules (Lung-RADS category 4A) and very suspicious nodules (Lung-RADS category 4B & 4X) should be more closely monitored in 3 and 1 month, respectively. A clinical comprehensive evaluation is required to determine whether the nodule is more likely benign or malignant, based on the type and size of the nodule, as well as medical record and laboratory tests (e.g., genetic and biomarker analysis). Prior to surgery, biopsy and preoperative localization is usually conducted. The standard surgical treatments include lobectomy or sublobar resections, while video-assisted thoracoscopic surgery (VATS) is a less-invasive surgical method. For those who are unfit or unwilling to receive surgical treatments, non-surgical treatments such as stereotactic body radiation therapy (SBRT) and radiofrequency ablation (RFA) may be the options. After treatment, physicians need to follow-up the patient every 3 months after treatment for 2 years, with CT repeated every 3–6 months.

Our review suggested the knowledge gap in the field and discussed the predictive value of GGNs and clinical decision-making for managing GGN, aiming to improve the diagnostic accuracy using a number of non-invasive tests in association with imaging scans (Figure 1). Future research should focus on validation of these non-invasive tests for clinical use in prediction and early diagnosis of different types of GGNs. In addition, these non-invasive approaches might also provide significant insight on understanding molecular profile and mechanisms of progression from GGN toward lung cancer, which may allow the opportunity for the development of novel and optimized approaches to add on predictive and diagnostic values of pulmonary GGNs for lung cancer.

## Author contributions

YD, CH, and JT drafted the manuscript. JT planned, supervised the work and was responsible for the integrity of the work as a whole. XZ and SX did critical revision of the manuscript for important intellectual content. All authors edited the manuscript and approved the submitted version.

## Footnotes

1
https://www.acr.org/-/media/ACR/Files/RADS/Lung-RADS/LungRADSAssessmentCategoriesv1-1.pdf
